# Longitudinal analysis of the impact of smoking exposure on atopic indices and allergies in early childhood

**DOI:** 10.1016/j.waojou.2023.100802

**Published:** 2023-07-22

**Authors:** Yi-Wen Wang, Kuo-Wei Yeh, Jing-Long Huang, Kuan-Wen Su, Ming-Han Tsai, Man-Chin Hua, Sui-Ling Liao, Shen-Hao Lai, Chih-Yung Chiu

**Affiliations:** a Department of Pediatrics, Chang Gung Memorial Hospital at Linkou, Chang Gung University, Taoyuan, Taiwan; b Department of Pediatrics, New Taipei Municipal TuCheng Hospital, Chang Gung Memorial Hospital and Chang Gung University, Taiwan; c Community Medicine Research Centre, Chang Gung Memorial Hospital, Keelung, Taiwan; d Department of Pediatrics, Chang Gung Memorial Hospital at Keelung, and Chang Gung University College of Medicine, Taoyuan, Taiwan; e Division of Pediatric Pulmonology, Chang Gung Memorial Hospital at Linkou, College of Medicine, Chang Gung University, Taoyuan, Taiwan

**Keywords:** Asthma, Cotinine, Eczema, Immunoglobulin E, Smoking

## Abstract

**Background:**

Exposure to smoking is recognized as a health hazard; however, a longitudinal analysis of the impact of smoking exposure in families on the allergic reactions related to childhood atopic diseases has not been well addressed.

**Methods:**

Children who completed a three-year follow-up period from the birth cohort were included in this study. The history of smoking exposure was recorded, and the urine cotinine levels were measured at 1 and 6 months, and 1, 2, and 3 years of age. Specific IgE levels against food and mite allergens were measured at age 6 months, and 1, 2, and 3 years. Their relevance to family smoking exposure and the subsequent development of atopic diseases was also analyzed. This study was approved by the Ethics Committee of Chang Gung Memorial Hospital (No. 102-1842C)

**Results:**

A total of 198 infants were enrolled in this study. The prevalence of passive smoking exposure among these children was as high as 45%. The urine cotinine levels were significantly higher in children with history of smoking exposure (*P* < 0.001). At 6 months of age, the food-specific IgE levels and the prevalence of eczema were significantly higher in children with smoking exposure than in those without smoking exposure (*P* < 0.05). By contrast, the urine cotinine levels were significantly higher in children with IgE sensitization (>100 kU/L, *P* < 0.05) at 3 years of age, which was also significantly associated with a higher prevalence of allergic rhinitis and development of asthma (*P* < 0.01).

**Conclusion:**

Family smoking exposure appears to be strongly associated with food sensitization in infancy and with IgE production in later childhood. This could potentially increase the susceptibility of developing infantile eczema and subsequent childhood airway allergies.

## Introduction

Smoking exposure is known as one of the most common public health concerns globally. Many studies have reported that smoking exposure leads to a multitude of health problems including cardiovascular and pulmonary diseases, as well as atopic diseases in adults.^1^, ^2^, ^3^ However, the impact of smoking exposure on allergic reactions and airway diseases in children has received less attention and remains uncertain.

IgE which specifically recognizes allergens and mediates immune responses, plays a significant role in allergic reactions. In children, IgE levels are recognized as a biomarker for predicting the development of allergies.^4^ Elevated serum total IgE levels have been reported in subjects exposed to smoking in several studies.^5^^,^^6^ However, the association between smoking exposure, total serum IgE levels, allergen-specific IgE levels, and atopic diseases has not been thoroughly investigated through longitudinal analysis in early childhood.

The major aim of this study was to explore the relationship between smoking exposure, cotinine levels, and allergic indices in children at 6 months, and 1, 2, and 3 years of age from a birth cohort in the Prediction of Allergies in Taiwanese Chinese (PATCH) study. The relevance of these factors to the risk of atopic diseases was also examined.

## Materials and methods

### Patients and data collection

A total of 198 children who completed a 3-year follow-up period in a birth cohort study launched at Chang Gung Memorial Hospital were enrolled. Detailed descriptions of subject recruitment have been previously reported.^7^ Information regarding demographic characteristics such as child's sex, breastfeeding history, household income, and history of family atopy was collected. A questionnaire survey was made to gather passive smoking exposure history of children. The questionnaire was filled in by parents or caregivers, which inquired about smokers living with the children and exposure to tobacco smoke.^8^ Informed consent of all subjects written in this study was obtained from the parents or guardians, and all experiments were performed in accordance with the relevant guideline. This study was approved by the Ethics Committee of Chang Gung Memorial Hospital (No. 102-1842C).

### Measurement of urine cotinine levels

Spot urine samples were subsequently collected at 1 and 6 months, and 1, 2, and 3 years of age. Urine cotinine levels were measured using a cotinine ELISA kit (Abnova, Cat#: KA0930), and were normalized to creatinine levels to minimize the differences in urinary concentration between samples.^8^

### Measurement of total serum IgE and allergen-specific IgE levels

Serum samples for total and allergen-specific IgE levels were collected and measured at 6 months, and 1, 2, and 3 years of age. As described in our previous study,^9^ total serum IgE level was measured by ImmunoCAP (Phadia, Uppsala, Sweden) and a commercial assay (ImmunoCAP Phadiatop Infant; Phadia) was used to measure specific IgE level of 2 most common food allergens, egg white and milk, and inhalant allergens including *D. pteronyssinus* and *D. farinae*. Allergen sensitization was defined as an allergen-specific IgE level ≥0.35 kU/L.

### Diagnosis of atopic diseases

The validated International Study of Asthma and Allergies in Childhood (ISAAC) questionnaire was used to collect information on allergic symptoms.^10^ The diagnostic criteria of atopic diseases including eczema, asthma, and allergic rhinitis were listed as follows; and the final diagnoses were made by the same pediatric pulmonologists at outpatient clinics.^9^^,^^11^, ^12^, ^13^, ^14^ Eczema was defined as a chronic relapsing course of pruritic rash over the face and/or extremities. Based on the Global Initiative for Asthma (GINA) guidelines, asthma was diagnosed with the presence of a recurrent wheeze or current use of asthma medication. The diagnosis of allergic rhinitis was defined as a history of nasal allergy symptoms, such as sneezing, nasal congestion, itching, or rhinorrhea or if the subject was currently using medication for these symptoms.

### Statistical analysis

Differences and comparisons in demographic characteristics between groups were analyzed using chi-square test, Student's t-test, or Mann–Whitney *U* test. Univariate and multivariate logistic regression analysis was used to study the associations between atopic diseases, smoking exposure, and their confounding factors. The odds ratios (OR) of each factor and corresponding 95% confidence intervals (CI) were also determined. Statistical Package for the Social Sciences (SPSS Statistics for Windows Version 20.0; Armonk, NY, USA) software was used for statistical analysis. Data were represented graphically by GraphPad Prism software (GraphPad Software Inc. Version 5.01; San Diego, CA, USA). All tests were two-tailed with a significance level of *P* < 0.05.

## Results

### Patient characteristics associated with smoking exposure

**Fig. 1:**
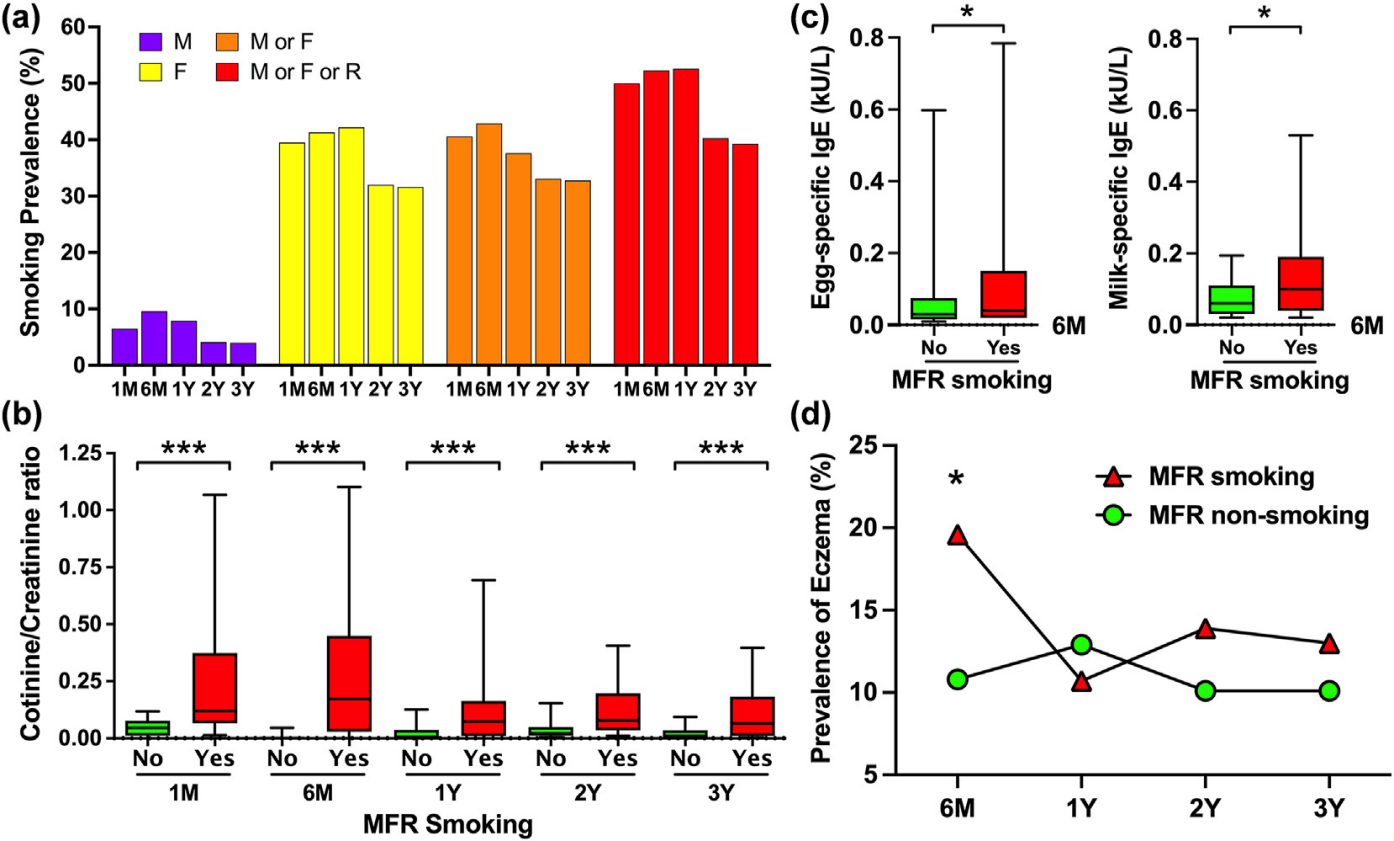
The prevalence of smoking exposure from different family members (a), and their relevance to the urine cotinine to creatinine ratio in children with and without smoking exposure (b) at 1 and 6 months, and 1, 2, and 3 years of age. Comparisons and differences between smoking exposure and food-specific IgE levels at 6 months of age (c), and the prevalence of eczema at different ages (d). M: mother; F: father; R: relatives; MFR: mother or father or relatives. ∗*P* < 0.05; ∗∗∗*P* < 0.001

The prevalence rates of smoking exposure in children from their mother, father, mother or father, and mother or father or relatives (MFR) were approximately 5%, 35%, 35%, and 45%, respectively (Fig. 1a). A nearly 10% decrease in smoking exposure was observed after the child turned 1 year old. Passive smoking refers to inhaling tobacco smoke, and smoking exposure to MFR can represent children's exposure to smoke. Therefore, MFR smoking was used as the dependent variable for further sub-analyses. Table 1 shows the baseline characteristics of 198 children categorized by MFR smoking exposure at 6 months and 1, 2, and 3 years of age. Compared with children without smoking exposure, there was a significantly lower household incomes in children with MFR smoking exposure at 6 months, and 1, and 2 years of age. Furthermore, a significant higher prevalence of formula feeding was observed in children with MFR smoking exposure at 1 and 2 years of age (*P* < 0.05).

**Table 1:** Baseline characteristics of 198 children in relation to smoking exposure to MFR at 6 months, and 1, 2, and 3 years of age.

	6 M			1Y			2Y			3Y		
	MFR smoking			MFR smoking			MFR smoking			MFR smoking		
Age	(+), n = 102	(−), n = 93	*P*	(+), n = 103	(−), n = 93	*P*	(+), n = 94	(−), n = 102	*P*	(+), n = 77	(−), n = 119	*P*
Family												
Maternal atopy	47(46.1%)	37(39.8%)	0.343	44(42.7%)	40(43.0%)	0.986	40(42.6%)	43(42.2%)	0.951	30(39.0%)	53(44.5%)	0.455
Paternal atopy	56(54.9%)	50(53.8%)	0.814	55(53.4%)	51(54.9%)	0.898	53(56.4%)	52(51.0%)	0.442	45(58.4%)	60(50.4%)	0.254
Older siblings	48(47.1%)	38(40.9%)	0.384	47(45.6%)	40(43.0%)	0.712	44(46.8%)	42(41.2%)	0.463	36(46.8%)	50(42.0%)	0.547
Household income												
Low (<500,000 NTD)	47(46.1%)	24(25.8%)	**0.011**	50(48.5%)	22(23.7%)	**0.001**	43(45.7%)	28(27.5%)	**0.028**	34(44.2%)	37(31.1%)	0.168
Medium (500,000–1,000,000 NTD)	38(37.3%)	50(53.8%)		35(34.0%)	53(57.0%)		36(38.3%)	52(51.0%)		30(39.0%)	58(48.7%)	
High (>1,000,000 NTD)	16(15.7%)	19(20.4%)		17(16.5%)	18(19.4%)		14(14.9%)	21(20.6%)		12(15.6%)	23(19.3%)	
Infant												
Sex, male (%)	59(57.8%)	45(48.4%)	0.383	60(58.3%)	48(51.6%)	0.351	56(59.6%)	53(52.0%)	0.284	44(57.1%)	65(54.6%)	0.729
Maternal age (Y)	30.2 ± 4.6	31.2 ± 4.0	0.111	30.1 ± 4.9	31.2 ± 3.9	0.097	30.2 ± 4.7	31.2 ± 4.0	0.233	30.4 ± 4.7	30.9 ± 4.1	0.437
Gestational age (wk)	38.1 ± 2.1	38.1 ± 1.6	0.461	38.2 ± 2.1	38.0 ± 1.7	0.275	38.1 ± 2.2	38.1 ± 1.5	0.455	38.1 ± 2.2	38.1 ± 1.7	0.954
Birth body weight (kg)	3.1 ± 0.5	3.1 ± 0.4	0.444	3.1 ± 0.5	3.0 ± 0.4	0.394	3.0 ± 0.6	3.1 ± 0.4	0.594	3.1 ± 0.5	3.1 ± 0.4	0.472
Birth body length (M)	0.5 ± 0.0	0.5 ± 0.0	0.489	0.5 ± 0.0	0.5 ± 0.0	0.577	0.5 ± 0.0	0.5 ± 0.0	0.069	0.5 ± 0.0	0.5 ± 0.0	0.340
Birth BMI (kg/m^2^)	12.3 ± 1.3	12.7 ± 2.7	0.464	12.3 ± 1.3	12.7 ± 2.7	0.393	12.5 ± 2.0	12.5 ± 2.2	0.145	12.4 ± 1.3	12.5 ± 2.5	0.239
Breastfeeding												
Exclusive	33(32.4%)	34(36.6%)	0.051	31(30.1%)	36(38.7%)	**0.009**	29(30.9%)	38(37.3%)	**0.024**	26(33.8%)	41(34.5%)	0.104
Partial	30(29.4%)	38(40.9%)		30(29.1%)	38(40.9%)		27(28.7%)	41(40.2%)		21(27.3%)	47(39.5%)	
Formula	39(38.2%)	21(22.6%)		42(40.8%)	19(20.4%)		38(40.4%)	23(22.5%)		30(39.0%)	31(26.1%)	

Data shown are mean ± SD or number (%) of patients as appropriate. BMI, body mass index; kg, kilogram; M, month; MFR, mother or father or relatives; wk, week; Y, year. All *P* values < 0.05, which is in bold, are significant.

### Association between MFR smoking exposure and urine cotinine levels

Fig. 1b shows the differences in the urine cotinine levels categorized by MFR smoking exposure at 1 and 6 months and 1, 2, and 3 years of age. Compared with children without smoking exposure, urine cotinine levels were significantly higher in children with MFR smoking exposure at all ages (*P* < 0.001).

### Association between MFR smoking exposure and the allergen-specific IgE levels and atopic diseases

The association between MFR smoking exposure and allergen-specific IgE levels was analyzed at 6 months, and 1, 2, and 3 years of age (Supplementary Fig. S1). In children with MFR smoking exposure, only egg- and milk-specific IgE levels were found to be significantly higher than those in children without smoking exposure at 6 months of age (*P* < 0.05) (Fig. 1c). Furthermore, a significantly higher prevalence of eczema was found in children with MFR smoking exposure at age 6 months (*P* < 0.05) (Fig. 1d). However, no significant associations between MFR smoking exposure and the prevalence of rhinitis and asthma at different ages were found.

### Association between MFR smoking exposure and the risk of atopic diseases

The univariate and multivariate logistic analyses of possible factors contributing to atopic diseases, including sex, family atopy history, having older siblings, MFR smoking, as well as its associated factors like household income and formula feeding were analyzed at different years of age. MFR smoking exposure was the most significant factor contributing to eczema at age 6 months (Table 2, *P* < 0.05). However, there was no association between MFR smoking and risk of rhinitis or asthma at different ages.

**Table 2:** Univariate and multivariate logistic regression analyses of factors associated with smoking exposure contributing to eczema at the age of 6 months.

Factors	OR	95% CI	*P*-value
Univariate			
Smoking exposure	2.320	0.993–5.418	0.052
House income	1.163	0.679–1.992	0.581
Breastfeeding	1.025	0.621–1.692	0.924
Sex, male	1.008	0.445–2.279	0.986
Maternal atopy	0.978	0.435–2.200	0.957
Paternal atopy	1.938	0.814–4.616	0.135
Older sibling	0.879	0.386–2.003	0.760
Multivariate			
Smoking exposure	2.575	1.058–6.267	**0.037**
House income			
Low	Reference		
Medium	1.048	0.395–2.782	0.925
High	1.527	0.487–4.786	0.467
Breastfeeding			
Exclusive	Reference		
Partial	1.474	0.535–4.059	0.453
Formula	1.039	0.350–3.085	0.945
Sex, male	1.019	0.433–2.400	0.966
Maternal atopy	0.956	0.401–2.277	0.919
Paternal atopy	1.955	0.782–4.884	0.151

CI, confidence interval; OR, odds ratio. All *P* values < 0.05, which is in bold, are significant.

### Association between urine cotinine levels and IgE sensitization and atopic diseases

**Fig. 2:**
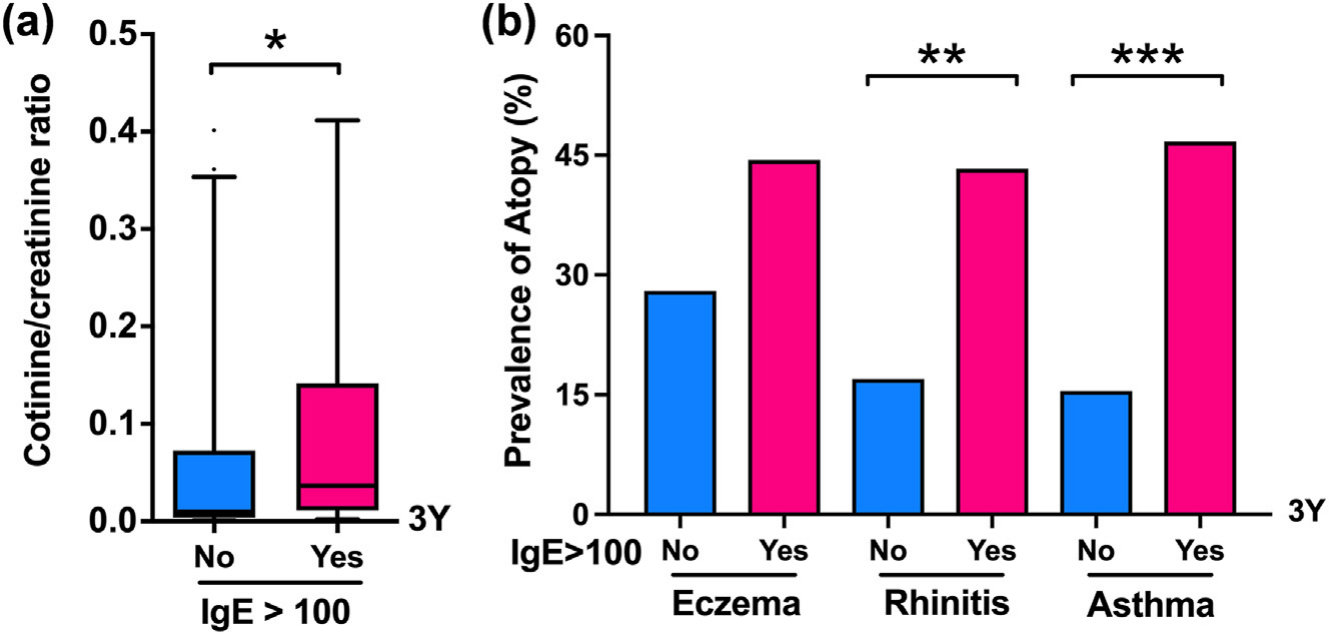
Comparisons and differences of IgE sensitization (>100 kU/L) with urine cotinine to creatinine ratio (a), and the prevalence of eczema, rhinitis, and asthma (b) at 3 years of age. IgE, immunoglobulin E. ∗*P* < 0.05; ∗∗*P* < 0.01; ∗∗∗*P* < 0.001

An analysis of the associations between urine cotinine levels, allergic sensitization, and atopic diseases was subsequently performed at different ages; however, significant higher urinary cotinine levels were found only in children with IgE sensitization at the age of 3 years compared with those without IgE sensitization (Fig. 2a, *P* < 0.05). Fig. 2b shows the association between IgE sensitization and the prevalence of atopic diseases at age 3, at which age a higher prevalence of allergic rhinitis and asthma but not eczema was significantly associated with IgE sensitization (*P* < 0.01).

## Discussion

Smoking exposure has been widely recognized as a health issue that leads to several diseases. However, its role in allergic reactions relevant to atopic diseases in childhood remains uncertain. This study provides a longitudinal overview of the dynamic relationships between family smoking exposure and allergen sensitization related to atopic diseases in early childhood.

Passive smoking exposure in children, resulting from parental smoking, is a global public health problem. In this study, the smoking prevalence of mothers and fathers was around 5% and 35% respectively, which is in accordance with a previous study in Taiwan.^15^ However, passive smoking exposure is common in children, with a prevalence rate as high as 45% in this study, indicating an underestimated exposure risk to smoking within the family. Most importantly, an approximately 10% decrease in smoking prevalence 1 year after childbirth appears to be related to the anti-smoking legislation of the government for smoking cessation during this experimental period but not protection from smoking for children.^15^^,^^16^ This finding suggests that there is still insufficient attention given to the impact of cigarette smoking on children's health within the family.

Smoking prevalence is associated with many factors such as physical conditions, environmental upbringing and socioeconomic status.^17^^,^^18^ One study has reported that lower household income was associated with a higher risk of smoking among household members.^19^ Furthermore, mothers with higher family income and higher education level were more likely to breastfeed,^20^ supporting the observation of a strong association of MFR smoking with household income and formula feeding in this study.

Serum cotinine level has been reported to be a reliable and helpful epidemiological marker of nicotine intake and environmental smoking exposure.^21^, ^22^, ^23^ In contrast to serum levels, urine cotinine levels also appeared to strongly correlated with MFR smoking exposure in this study. In clinic, collecting urine samples is a relatively simple, non-invasive, and safe method for children.^24^ This finding indicates that cotinine levels determined in urine could be a useful biomarker for MFR smoking exposure in early childhood.

Passive smoking exposure is associated with allergic IgE production and atopic diseases in children.^5^^,^^25^ Recent studies mentioned that passive exposure to smoking from infancy increases the risk of food allergy and eczema in childhood,^26^^,^^27^ supporting the finding of a strong association between MFR smoking exposure and food-specific IgE levels and risk of infantile eczema in this study. MFR smoking exposure appears to be an important factor contributing to food sensitization and eczema in infancy.

In later childhood, several studies have reported a link between exposure to smoking, IgE sensitization and urine cotinine levels.^5^^,^^25^ Clinically, IgE has been shown as a strong predictor of the outcome of childhood allergic rhinitis and asthma.^26^ In this study, despite the fact that there was no association between MFR smoking exposure and allergic diseases, a strong association between IgE sensitization and urinary cotinine levels related to MFR smoking exposure indicates that an increases in total serum IgE levels associated with MFR smoking exposure may increase susceptibility to allergic airway diseases in later childhood.

Limitations of this study include the relatively small sample size of 198 subjects and limited statistical power to detect the association for sub-analyses. Nevertheless, the strength of this study is its longitudinal design, which ensures regular follow-up of subjects to get consequent data of smoking exposure, urine samples for cotinine levels, blood samples for allergen-specific IgE levels, and make a faithful diagnosis of atopic disease at outpatient clinics. Most importantly, the fact that this study originates from a birth cohort makes the results demonstrated here valid and potentially important.

In conclusion, passive smoking exposure is common among children with a prevalence rate as high as 45%. Urine cotinine levels appear to be a reliable biomarker for smoking exposure in families. A strong association of family smoking exposure with food sensitization and risk of infantile eczema indicates that smoking exposure within family is an important factor for allergy and eczema in infancy. By contrast, an increase in IgE production associated with exposure to smoking in family may increase susceptibility to allergic airway diseases in later childhood.

## Abbreviations

CI, confidence intervals; ISAAC, International Study of Asthma and Allergies in Childhood; MFR, mother or father or relatives; OR, odds ratio; PATCH, Prediction of Allergies in Taiwanese Chinese study.

## Acknowledgments

We would like to express our gratitude to the participants of our study, the pediatricians involved in the recruitment of these participants, as well as the entire PATCH team, including interviewers, nurses, technicians, and research assistants.

## Funding

This study was supported by research grants of CMRPG3K1361-2 and CMRPG3L1421-2 from the 10.13039/100012553Chang Gung Memorial Hospital, Taiwan and 10.13039/501100004663MOST 109-2314-B-182-075-MY2 of the Ministry of Science and Technology in Taiwan.

## Data availability

All the data are included in this paper.

## Author contributions

Y.-W.W. drafted and revised the manuscript. K.-W.Y., J.-L.H., and K.-W.S. performed experimental work and interpretation. M.-H.T., M.-C.H., S.-L.L., and S.-H.L. were responsible for clinical evaluation of the children and data collection. C.-Y.C. design and supervised the study. All authors discussed the results and approved the final manuscript.

## Ethics approval

This study was approved by the Ethics Committee of CGMH (No. 102-1842C).

## Consent for publication

All authors have agreed with this publication in the World Allergy Organization Journal.

## Declaration of competing interest

All the authors declare no conflicts of interest in relation to the present study.
