# Supplementary material for: Longitudinal analysis of the impact of smoking exposure on atopic indices and allergies in early childhood
Source: World Allergy Organ J. 2023 Jul 22;16(7):100802. doi: 10.1016/j.waojou.2023.100802 (PMC10374959; doi:10.1016/j.waojou.2023.100802)
Supplement: Multimedia component 1 [file mmc1.pdf]

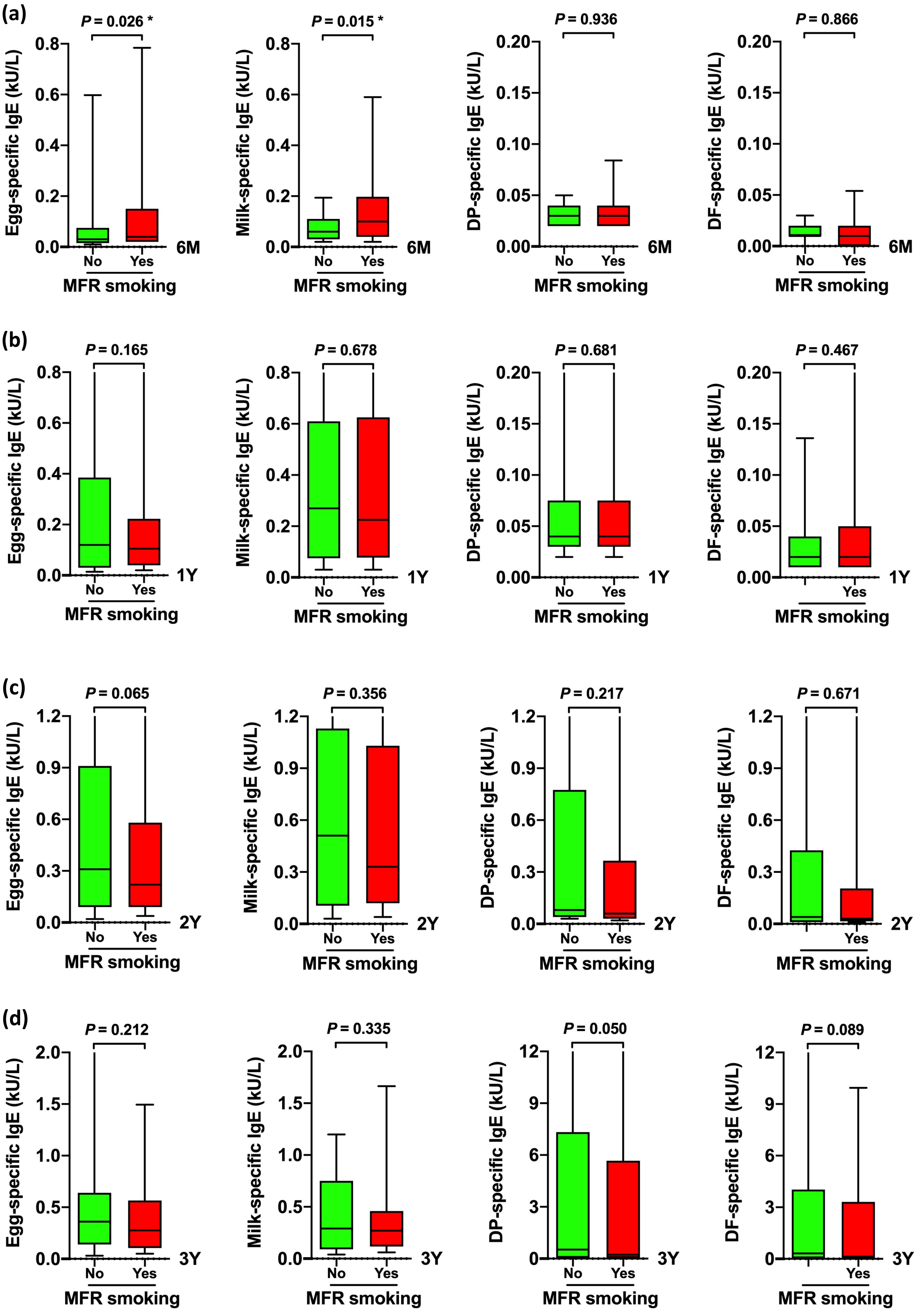

Supplementary Figure S1: Comparisons and differences between smoking exposure and allergen-specific IgE levels at 6 months (a), and 1 (b), 2 (c), and 3 (d) years of age. DP: *Dermatophagoides pteronyssinus*; DF: *Dermatophagoides farinae*.
